# Epigenetics in myeloid derived suppressor cells: a sheathed sword towards cancer

**DOI:** 10.18632/oncotarget.10767

**Published:** 2016-07-21

**Authors:** Chao Zhang, Shuo Wang, Yufeng Liu, Cheng Yang

**Affiliations:** ^1^ Department of Urology, Zhongshan Hospital, Fudan University, Shanghai, China; ^2^ Department of Plastic Surgery, Zhongshan Hospital, Fudan University, Shanghai, China; ^3^ Shanghai Key Laboratory of Organ Transplantation, Shanghai, China; ^4^ Department of Nursing, General Surgery, Zhongshan Hospital, Fudan University, Shanghai, China

**Keywords:** epigenetics, myeloid derived suppressor cell (MDSC), microRNA (miRNA), small interfering RNA (siRNA), DNA methylation

## Abstract

Myeloid-derived suppressor cells (MDSCs), a heterogeneous population of cells composed of progenitors and precursors to myeloid cells, are deemed to participate in the development of tumor-favoring immunosuppressive microenvironment. Thus, the regulatory strategies targeting MDSCs' expansion, differentiation, accumulation and function could possibly be effective “weapons” in anti-tumor immunotherapies. Epigenetic mechanisms, which involve DNA modification, covalent histone modification and RNA interference, result in the heritable down-regulation or silencing of gene expression without a change in DNA sequences. Epigenetic modification of MDSC's functional plasticity leads to the remodeling of its characteristics, therefore reframing the microenvironment towards countering tumor growth and metastasis. This review summarized the pertinent findings on the DNA methylation, covalent histone modification, microRNAs and small interfering RNAs targeting MDSC in cancer genesis, progression and metastasis. The potentials as well as possible obstacles in translating into anti-cancer therapeutics were also discussed.

## CANCER IMMUNOTHERAPY TARGETING MDSCs

Cancer immunotherapies have become research highlights and shown promising effect in multiple pre-clinical studies [1-3]. However, the clinical outcomes are usually unsatisfactory as a result of relatively weak potency of these interventions *in vivo* [4]. One explanation is that several distinct subsets of tumor-infiltrating myeloid cells with immunosuppressive function, named as myeloid derived suppressor cells (MDSCs), constitute immune tolerant microenvironment which ameliorates or even abrogates the efficacy of immunotherapies [5, 6].

### MDSCs and their subsets

MDSCs are a heterogeneous population of cells generally composed of progenitors and precursors to dendritic cells, macrophages and granulocytes at various stages of differentiation [7, 8]. In physiological conditions, these immature myeloid cells (IMCs) migrate into peripheral lymphoid organs and eventually differentiate into mature dendritic cells, macrophages or granulocytes. Both endogenous and exogenous pathological stresses, however, can inhibit the differentiation of IMCs while promote expansion of this population. IMCs subsequently become activated by tumor-derived factors and host cytokines, resulting in the generation of MDSCs with potent immunosuppressive capacity [9]. In mice, MDSCs are uniformly identified by co-expression of surface markers CD11b and Gr-1, but with two subtypes based on their distinct expression of Ly-6C and Ly-6G [10]. The CD11b^+^Ly6G^+^Ly6C^low^ cells, called G-MDSCs, are demonstrated to have a granulocytic phenotype and express high levels of reactive oxygen species (ROS) but only nominal amounts of nitric oxide (NO). G-MDSCs exert immunosuppressive function via ROS-mediated mechanisms in a cell contact dependent manner [10]. To be specific, peroxynitrite produced by G-MDSCs leads to the nitration of the T-cell common receptors (TCRs) and CD8 molecules, which interfere the specific binding of antigen peptide to TCRs and renders them unresponsive to antigen-specific stimulation. However, T cells still maintained their responsiveness to nonspecific stimuli [11]. In contrast, the CD11b^+^Ly6G^-^Ly6C^high ^cells, called M-MDSCs, present a monocytic-like morphology and exert immunosuppressive function via high expression of inducible nitric oxide synthase (iNOS) and arginase-1 following the activation of STAT3 signaling in a cell contact independent manner [10]. The increased activity of arginase-1 leads to enhanced L-arginine catabolism and depletes this non-essential amino acid in the microenvironment. The paucity of L-arginine inhibits T-cell proliferation through several different mechanisms, including decreasing their CD3ζ expression [12] and preventing their upregulation of the expression of the cell cycle regulators cyclin D3 and cyclin-dependent kinase 4 (CDK4) [13]. NO is able to inhibit the downstream pathway of IL-2 receptor by blocking the phosphorylation of signaling proteins (like Jak3 or Stat5) [14] or to induce T cell apoptosis directly [15]. Both of these two subsets can express pro- and anti-inflammatory mediators [16-18]. Unlike murine MDSCs, the human MDSCs are ambiguously defined owing to the lack of specific markers. The human MDSCs are commonly defined as CD11b^+^CD33^+^HLA-DR^low/- ^cells [19]. Some investigators affirmed that human MDSCs could also be subdivided into two main subsets: CD15^+^CD14^-^CD11b^+^CD33^+^HLA-DR^low/- ^G-MDSCs and CD15^-^CD14^+^CD11b^+^CD33^+^HLA-DR^low/-^ M-MDSCs, but with no agreement to date [20].

### MDSCs promote tumor progression

MDSCs are reported to involve in a large variety of disorders such as infectious diseases [21], inflammation [22], autoimmune diseases [23], organ transplantation [24] and more importantly to mention, in tumors [25]. Plenty of evidences indicate that MDSCs accumulate in the tumor site not only in cancer patients but also in transplanted or spontaneous tumor-bearing animal models [25-28]. MDSCs have capacity to support tumor growth and metastasis through remodeling of the tumor microenvironment [29]. In addition to suppress tumor antigen-driven activation of T cells [30], they have been shown to produce vascular endothelial cell growth factor (VEGF), β-fibroblast growth factor (β-FGF), VEGF analogue Bv8, and matrix metalloproteinase 9 (MMP9), all essential mediators of angiogenesis and tissue invasion at the tumor site [31-33]. The expression of these mediators has been linked to MDSC-mediated tumor progression and is independent of their immunosuppressive capacity [34]. Thus, the efficient inhibition of MDSC's expansion, accumulation, migration and function has the potential to reform the tumor microenvironment and make it benefit anti-tumor immunotherapeutic strategies. Recent studies have seen epigenetic modification of MDSCs as a promising tool to achieve this goal. Epigenetics defines all heritable modulations in gene expression but without any alterations in the DNA sequence itself [35]. These epigenetic modifications enable significant flexibility in gene expression, rather than just turning them “ON” or “OFF”. Three systems, including DNA modification, histone modification and RNA-associated interference, are used to initiate and sustain epigenetic silencing [36-39]. We reviewed the recent literature on epigenetic modulations of MDSCs, including DNA methylation and histone modification of target genes and post-transcriptional regulation with RNA interference.

## DNA METHYLATION IN MDSCs' GENES

DNA methylation, one of the most important forms of epigenetic modification, inhibits gene expression with transcription machinery: Once DNA is methylated, transcriptional factors are blocked from gaining access to the gene, and thus expression is effectively silenced. Both *de novo* and inherited DNA methylation is dependent on DNA methyltransferases (DNMTs) [40], which transfer methyl groups to the 5-position on the cytosine residue found in CpG clusters within DNA sequence [41] . To be specific, DNMT1 binds preferentially to hemi-methylated DNA and is considered to be the maintenance DNMT, while the DNMT3 family, 3a and 3b, is considered to be responsible for *de novo* methylation[42].

### Δ9-Tetrahydrocannabinol mediated DNA methylation

Δ9-Tetrahydrocannabinol (THC), an exogenous cannabinoid derived from the *Cannabis sativa* plant, is validated to be potent inducers of MDSCs [43]. In fact, administration of THC into wild type mice caused increased methylation at the promoter region of DNMT3a and DNMT3b in THC-induced MDSCs, resulting in reduced expression of DNMT3a and DNMT3b [44].Therefore, promoter region methylation was decreased at arginase-1 and STAT3 in THC-induced MDSCs, and consequently, these two genes were actively transcribed in MDSCs. The high expression of arginase-1 and STAT3 led to enhanced accumulation of MDSCs in cancer models and increased suppressive function [44]. In addition, THC-induced MDSCs expressed high levels of S100A8, which is essential for the enhanced suppressive function triggered by THC. All in all, this study revealed that THC mediates epigenetic changes to promote MDSC differentiation and function and that S100A8 plays a critical role in this process [44].

## COVALENT HISTONE MODIFICATION IN MDSCs' GENES

Covalent histone modification, another form of epigenetic regulation, refers to the process in which covalent bonds alter the core structure of histones and influence the binding of ‘effector’ molecules to DNA sequences, therefore affecting patterns of gene expression[45, 46]. These covalent modifications include lysine and arginine acetylation, serine and threonine phosphorylation, ubiquitination and other underappreciated modifications [47]. Histone acetylation is hitherto the best-studied histone modification. Levels of acetylation of the core histones result from the dynamic balance between the opposing activities of histone acetyltransferases (HATs) and histone deacetylases (HDACs) [38].

### Histone deacetylase inhibition by TSA

Histone acetylation and deacetylation modulate gene expression in a mutually antistatic way. In the process of histone deacetylation, histone deacetylases (HDACs) are recruited to the gene promoters by transcriptional factors or co-repressors, where they block gene accessibility and transcription. HDAC inhibition enables to increase the extent of histone acetylation, resulting in tighter DNA binding and reduction in gene expression [48]. TSA is a naturally occurring antifungal metabolite produced by Streptomyces and has shown potently HDAC-inhibiting activity in a variety of researches [49]. Rosborough et al. demonstrated that exposure of GM-CSF stimulated murine bone marrow cells to TSA elicited a robust expansion of monocytic MDSC (CD11b^+^Ly6C^+^F4/80^int^CD115^+^) *in vivo* and *in vitro*, which suppressed allogeneic T cell proliferation in a NOS- and heme oxygenase (HO)-1-dependent manner [50].

### Histone deacetylase 11 (HDAC11)

HDAC11 is the newest member of the histone deacetylase family and has been reported to involve in hematopoietic lineage differentiation, as well as graft *versus* host disease (GVHD) [51]. In the study by Sahakian et al., HDAC11 seemed to function as a negative regulator of MDSC expansion and function *in vivo* [52]. The transition of immature myeloid cells to MDSCs required a decrease in the expression of HDAC11, indicating that HDAC11 served as a gate-keeper of myeloid differentiation [52]. Tumor-bearing HDAC11-knockout mice (HDAC11-KO) presented a more suppressive MDSC population and enhanced tumor growth kinetics when compared to the wild-type mice [52]. Considering the negative role of HDAC11 in MDSC expansion and function, expanding this epigenetic modifier may function as a powerful strategy to efficacious immunotherapies.

### Histone deacetylase 2 (HDAC2)

In cancer, G-MDSC is the dominant subpopulation of MDSC that accumulated in tumor microenvironment[53]. It is interesting to find that a large amount of M-MDSCs could acquire the phenotypic, morphological and functional features of G-MDSCs in tumor-bearing mice [54]. Further experiments indicated that the inhibition of Rb1, a member of the Rb family of transcriptional regulators that control cellular proliferation and differentiation, played a key role in regulation of M-MDSC differentiation towards G-MDSC. HDAC2 can directly bind to rb1 promoter and lead to silencing of rb1 expression [54]. So epigenetic modifications mediated by HDAC2 promoted the phenotype switch from M-MDSC to G-MDSC in cancer by transcriptional silencing of Rb1 gene. These findings showed a novel way for the selective therapeutic targeting of these cells in cancer.

## POST-TRANSCRIPTIONAL REGULATION OF MDSCs WITH MIRNAs

Post-transcriptional regulation is a highly conserved biological phenomenon, in which microRNAs (miRNAs) specifically recognize and degrade a homologous host mRNA, leading to the targeted gene being silenced[55]. This process is acknowledged as post-transcriptional gene silencing (PTGS)[56-58]. MicroRNAs (miRNAs) serve as important tools to implement RNA interference. They are small non-coding RNAs of 19-25 nucleotides in length that naturally exist in almost all eukaryotes ranging from trypanosome to human being[59]. In physiological conditions, miRNAs act as regulators of genes expression involved in fundamental cell processes such as development, differentiation and death[59]. Emerging studies have recently identified the vital role of miRNAs in the expansion, development, migration and function of MDSCs, which facilitate tumor cells evading from immune surveillance.

### miR-210

Noman et al. demonstrated hypoxia-inducible factor-1a (HIF1α)-induced over expression of miR-210 potentiated MDSC's tumor-promoting function by increasing arginase activity and NO production [60]. In tumor MDSC, HIF1α was bound directly to a transcriptionally active hypoxia-response element in the miR-210 proximal promoter [60]. MiR-210 increased mRNA and protein levels of arginase-1, IL-16, and chemokine C-X-C motif ligand 12 (CXCL12) in MDSCs. Overexpression of miR-210 strengthened MDSC-mediated T-cell suppression *in vivo* [60]. These results establish a new link between miR-210 and MDSC-mediated immune suppression under hypoxia in the tumor microenvironment and implicated the use of miR-210 inhibitor oligonucleotide as adjuvant tool for boosting the immune system in cancer patients.

### miR-9

Tian et al. reported in their study that miR-9 could inhibit the differentiation and promote immunosuppressive function of MDSCs [61]. They found that inhibition of miR-9 promoted the differentiation of MDSCs with significantly reduced immunosuppressive function whereas overexpression of miR-9 markedly enhanced the function of MDSCs *in vitro* study. MiR-9 performed on MDSCs differentiation by targeting runt-related transcription factor 1 (Runx1), an essential transcription factor in regulating MDSC differentiation and function [61]. In Lewis rat with lung carcinoma, knockdown of miR-9 significantly impaired the activity of MDSCs and prohibited the tumor growth. The clinical data also showed that high levels of miR-9 were observed in tumor tissues. Furthermore, miR-9 positively correlated with arginase whereas Runx1 negatively correlated with arginase [61]. This study indicated that inhibition or depletion of miR-9 could reduce MDSC-mediated suppression and benefit antitumor immunity, which might be further validated as a potential therapeutic target.

### miR-690

Hegde and his colleagues observed THC led to robust induction of functional MDSCs in mice [62]. They then performed a genome-wide analysis of miRNA expression by microarray-based profiling in highly immunosuppressive CD11b^+^Gr-1^+^ MDSCs induced by THC *in vivo* and identified differentially expressed miRNA unique to functional MDSCs including up-regulation of miR-690, miR-22, miR-15b and miR-27a and down-regulation of miR-324-5p, miR-335-5p. Altered miRNA expression regulated the target transcription factors and genes involved in MDSC activation, expansion and myeloid maturation [62]. Especially, miRNA-690 was highly overexpressed in THC-MDSCs. Transcription factor CCAAT/enhancer-binding protein (C/EBP) was identified as a potential functional target of miR-690 [62]. MiR-690 knockdown was able to unblock and significantly increase C/EBP expression establishing the functional link.

### miR-494

Liu et al. identified that miR-494, whose expression was dramatically induced by TGF-β1, as an essential player in increasing the accumulation and activity of MDSCs by targeting of phosphatase and tensin homolog (PTEN) and activation of the Akt pathway [63]. Expression of miR-494 not only enhanced CXCR4-mediated MDSC chemotaxis but also altered the intrinsic apoptotic/survival signal by targeting of PTEN, thus contributing to the accumulation of MDSCs in tumor tissues [63]. Knockdown of miR-494 significantly reversed the activity of MDSCs and inhibited the tumor growth and metastasis of 4T1 murine breast cancer *in vivo* [63]. Suppression of miR-494 not only generates anti-tumor immunity but also inhibits tumor metastasis and thus might be explored as a potential therapeutic target.

### miR155 and miR-21

Li et al. identified miR-155 and miR-21 as the two most upregulated miRNAs during the induction of MDSC from the bone marrow cells by GM-CSF and IL-6 [64]. Overexpression of miR-155 and miR-21 enhanced whereas depletion of miR-155 and miR-21 reduced the frequencies of cytokine-induced MDSC. Furthermore, miR-155 and miR-21 showed a synergistic effect on MDSC induction via targeting SHIP-1 and PTEN, respectively, leading to STAT3 activation. In addition, dexamethasone could strongly enhance MDSC expansion through upregulating miR-155 and miR-21 expression, and this effect was abolished by depleting cellular miR-155 and miR-21 [64].This study provided potential novel targets of miR-155 and miR-21 for controlling inflammation and autoimmune activity *in vivo*. Another study focused on the role of miR-155 in tumor promotion. It was illustrated that miR-155 accelerated the accumulation of functional MDSCs in the tumor microenvironment by suppressor of cytokine signaling (SOCS) 1 repression and reduced ability to license the generation of CD4^+^Foxp3^+^ regulatory T cells (Tregs), thereby facilitating tumor growth [65]. Host miR-155 deficiency promoted overall antitumor immunity.

### miR-17-5p and miR-20a

MDSCs transfected with miR-17-5p or miR-20a are less able to suppress Ag-specific CD4^+^ and CD8^+^ T cells both *in vitro* and *in vivo* [66]. In tumor-bearing mice, the expression of miR-17-5p and miR-20a in tumor-associated MDSCs was found to be lower than in Gr1^+^CD11b^+^ cells isolated from the spleens of disease-free mice, indicating that tumor-associated factor downregulated the expression of these two microRNAs, which contribute to immune tolerant microenvironment in tumor sites [66].

### miR-223

Liu et al. demonstrated that miR-223 could remarkably inhibit differentiation of bone marrow cells (BMCs) into CD11b^+^Gr1^+^MDSCs in the presence of tumor-associated factors by targeting myocyte enhancer factor 2C (MEF2C) *in vitro* [67]. In reconstituted tumor models, miR-223 also suppressed accumulation of MDSCs, whereas its targeting molecule MEF2C increased in accumulated MDSCs accordingly [67]. Besides this, tumor growth was slower in mice infused by miR223-engineered BMCs than in mice infused with control transfected BMCs [67]. These studies implied that the up-regulation of miR-223 in tumor-induced CD11b^+^ Gr1^+^ MDSCs may exert an important role in controlling the increased accumulation of MDSCs in patients with tumor.

### miR-146a

Although there were no evidences that miR-146a had direct influence on MDSCs' development, Boldin et al. reported that miR-146a, whose expression was up-regulated after immune cell maturation and/or activation, could inhibit the proliferation of multiple myeloid lineages, including CD11b+Gr-1+ population[68]. In fact, CD11b+GR1+blasts were demonstrated to be the major population of the expanding myeloid cells in miR-146a knock out mice. MiR-146a deficient mice bear higher risk of developing myeloid malignancies because of uncontrolled myeloid cell proliferation [69]. So it is possible that the overexpression of miR-146a could inhibit MDSCs' expansion and reduce tumorigenesis.

### miR-424

In humans, miR-424 up-regulation was associated with human monocyte/macrophage differentiation from CD34+ hematopoietic progenitors. The master transcription factor

PU.1 activated the transcription of miR-424 and suppressed NFI-A, an inhibitor of monocyte differentiation, thereby enhancing M-CSFr expression and monocytic differentiation [70]. Thus, up-regulation of miR-424 enabled to reduce MDSC population by promoting their differentiation into mature cells.

### miR-181b

Garzon et al. studied the role of miRNAs in granulopoiesis in acute promyelocytic leukemia (APL) patients during all-trans-retinoic acid (ATRA) treatment [71]. They found that miR-181b was downregulated after ATRA treatment while up-regulated in APL patients without treatment. The expression of miR-181b positively correlated with the active proliferation and accumulation of myeloid progenitors in humans [71].

### miR-34a

MiR-34a was able to induce MDSC expansion both in chimera and transgenic mice. Detailed study found that overexpression of miR-34a could inhibit MDSC apoptosis by suppressing the expression of N-myc but without affecting MDSC proliferation [72]. This study implied that down-regulation of miR-34a could reduce the number of infiltrated MDSCs in tumor by inducing apoptosis.

## THERAPEUTIC STRATEGIES ON MDSCs EPIGENETICS WITH SIRNA

Small interfering RNAs (SiRNAs) are artificial double-strand RNAs (dsRNAs) of 21-25 nucleotides in length that generate during PTGS and RNAi [73]. These small dsRNAs are made intentionally to serve as guide RNAs for target recognition and as a post-transcriptional regulator to effect on gene expression. Since the introduction of 21-nucleotide artificial siRNAs that triggered gene silencing in mammalian cells [74, 75], synthetic siRNAs have generated much interest in biomedical research. Here, artificial siRNAs targeting distinct MDSC genes have been used to exert an anti-cancer efficacy.

### A20 siRNA

A20 (also known as TNFAIP3) is originally identified as a primary TNF-α responsive gene in human umbilical vein endothelial cells (HUVEC) [76]. A20 gene encodes a 790-amino acid zinc finger protein [77], which negatively regulates inflammation, innate immunity and adaptive immunity. Shao et al. found that A20 was overexpressed in MDSCs [78]. The treatment of tumor-bearing mice with siRNA targeting A20 inhibited the growth of tumors. The infiltration of MDSCs was dramatically reduced after A20 siRNA treatment for that A20 siRNA induced MDSC apoptosis by elevating cleaved caspase-3 and caspase-8 level with the activation of JNK pathway [78]. Thus, this study suggested that A20 might be a potential target in anticancer therapy by inducing MDSC apoptosis in tumor microenvironment.

### STAT3 siRNA

In prostate cancer, the tumor-associated MDSCs potently inhibit autologous CD8+T cells proliferation and production of IFN-γ and granzyme-B, thereby impairing anti-tumor immunity. Hossain et al. previously generated an original strategy to silence genes specifically in toll-like receptor (TLR) 9 positive myeloid cells using CpG-siRNA conjugates and verified that human granulocytic MDSCs expressed TLR9 and rapidly internalized naked CpG-STAT3 siRNA, thereby silencing STAT3 expression [79, 80]. They also demonstrated that STAT3 blocking abrogated immunosuppressive effects of MDSCs on effector CD8^+^ T cells and these effects depended on reduced expression and enzymatic activity of arginase-1, a downstream STAT3 target gene and a potent T-cell inhibitor [81]. Disruption of STAT3 signaling in the tumor microenvironment with concurrent TLR9 stimulation has potential to elicit effective antitumor immune responses without toxicities associated with pharmacologic agents [81]. Consistently, STAT3 siRNA was also demonstrated to enhance anti-tumor immunity by abrogating MDSCs' suppressive function in head and neck squamous cell carcinoma (HNSCC) in another study [82]. They found MDSCs sorted from the tumors, draining lymph nodes, and peripheral blood of HNSCC patients showed high phosphorylated STAT3 levels that correlated with arginase-1 expression levels and activity. STAT3 could bind to the promoter region of arginase-1 to activate its transcription [82]. Thus, STAT3 siRNA alleviated MDSC immunosuppressive function by blocking STAT-3 triggering arginase-1 expression.

### Stem cell factor (SCF) siRNA

It is acknowledged that tumor-derived factors are involved in the accumulation of MDSCs and blockade of tumor factors can prevent T-cell anergy and Treg development [83-85]. Stem cell factor is one of such tumor factors that expressed by various human and murine carcinoma (e.g. melanoma, pancreatic cancer, colorectal carcinoma) [86-88]. Pan et al. demonstrated that mice bearing tumor cells with SCF siRNA exhibited significantly reduced MDSC expansion and restored proliferative responses of tumor-infiltrating T cells, leading to decreased tumor angiogenesis, decreased number of Foxp3^+^ Tregs, possibly suppressed Th2 responses and enhanced Th1 responses. Thus, SCF siRNA could improve immune therapy for the treatment of advanced tumors [89].

### Caseine kinase 2 (CK2) siRNA

Caseine kinase 2 (CK2) is one member of serine/threonine kinase family. Despite of its constitutive activation and ubiquitous expression in a variety of cell types and tissues, its overexpression was documented in the number of solid and hematologic malignancies [90, 91]. Cheng et al. reported that in mice injected with CT26 colon carcinoma, inhibition of CK2 by siRNA restored Notch signaling in MDSCs, substantially improving their differentiation and inhibiting their expansion both *in vitro* and *in vivo* without displaying signs of toxicity [92]. From this perspective, pharmacologic inhibition of CK2 may have value in an immunotherapeutic anticancer approach.

## PERSPECTIVE

**Figure 1:**
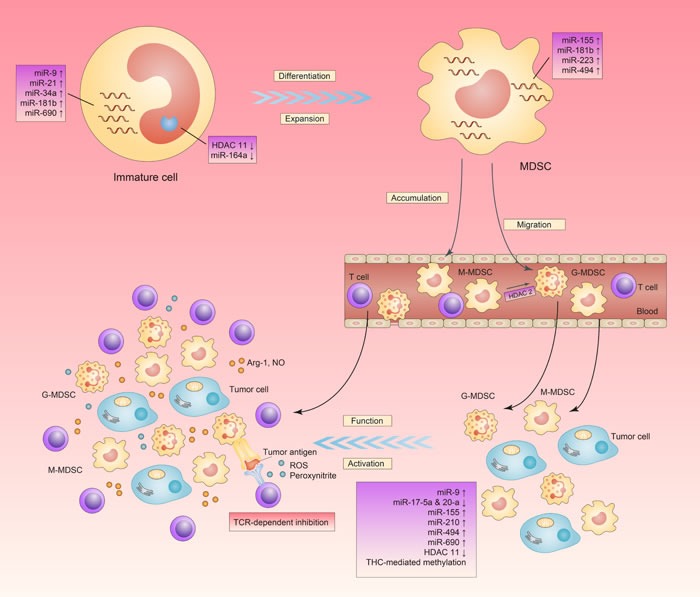
Effect of epigenetics on MDSC's expansion, differentiation, migration, activation and function This schematic represents all the biological behaviors of MDSCs from differentiating from immature cells to performing immunosuppressive function in tumor microenvironment. In each process, microRNAs and other epigenetic approaches play an either positive or negative role. In the tumor site, M-MDSCs inhibit T cell proliferation in a nitric oxide (NO) and arginase-1 (Arg-1) dependent way. G-MDSCs interfere the specific binding of antigen peptide to T-cell common receptors (TCRs) via inducing the nitration of TCRs, which renders T cells unresponsive to antigen-specific stimulation.

The highlighted researches rendered supports to the epigenetic modulation of MDSCs by DNA and histone modification, microRNA and siRNA serving as effective immunotherapeutic strategies for fighting against cancer (Table 1-2). These studies observed that epigenetic modulation on MDSCs could alter their expansion, differentiation, migration, activation and function, thereby influencing tumor growth, progress and metastasis despite of limited understanding of the molecular nature in the process (Figure 1). However, the following questions need to be answered before it can be eventually translated from bench to bedside.

**Table 1:** Summary of epigenetic regulation of myeloid derived suppressor cells (I)

Epigenetic modulation	Target gene or pathway	Effect on MDSCs	Disease/model	Species	Reference
miR-210	Arginase-1,CXCL12,IL-16	enhance immunosuppression	B16-F10 melanoma/4T1 mammary carcinoma cell inoculation	mice	[60]
miR-9	Runt-related transcription factor 1(Runx1)	enhance immunosuppression and promote differentiation	Lewis lung carcinoma cell inoculation/Lung carcinoma	mice/human	[61]
miR-494	Phosphatase and tensin homolog(PTEN)/Akt pathway	enhance migration and immunosuppression	4T1 mammary carcinoma cell/Lewis lung carcinoma/B16 melanoma/EG7 T lymphoma/A20 lymphoma/ CT26 colon carcinoma cell inoculation	mice	[63]
miR-690	Transcription factor CCAAT enhancer-binding protein ( C/EBPα)	regulate activation, expansion and maturation	EL-4 lymphoma cell inoculation	mice	[62]
miR-155	SOCS1/SHIP-1/ PTEN	promote accumulation and enhance immunosuppression	Lewis lung carcinoma/MC38 colon cancer cell inoculation	mice	[64],[65]
miR-21	SHIP-1/ PTEN	enhance the frequencies and induce expansion	Lewis lung carcinoma cell inoculation	mice	[64]
miR-17-5p and miR-20a	STAT3	alleviate immunosuppression	CT-26 colon carcinoma/Lewis lung carcinoma/1D8 ovarian carcinoma cell inoculation	mice	[66]
miR-223	Myocyte enhancer factor 2C (MEF2C)	suppress accumulation	CT-26 colon carcinoma/Lewis lung carcinoma/1D8 ovarian carcinoma cell inoculation	mice	[67]

**Table 2:** Summary of epigenetic regulation of myeloid derived suppressor cells (II)

Epigenetic modulation	Target gene or pathway	Effect on MDSCs	Disease/model	Species	Reference
miR-146a	IRAK1/TRAF6/NF-kB pathway	inhibit expansion	Immune deficiency	mice	[68, 69]
miR-424	PU.1/ NFI-A	promote differentiation	acute promyelocytic leukemia	human	[70]
miR-181b	CYLD, NF-kB	promote proliferation and accumulation	acute promyelocytic leukemia	human	[71]
miR-34a	N-myc	inhibit apoptosis and induce expansion	chimera	mice	[72]
A20 siRNA	A20	induce apoptosis and inhibit immunosuppression	EG7 T lymphoma/B16-F10 melanoma cell inoculation	mice	[78]
STAT3 siRNA	STAT3-arginase 1	abrogate immunosuppressive function	Head and neck squamous cell carcinoma (HNSCC)/prostate cancer	human	[80],[81]
SCF siRNA	Stem cell factor (SCF)	reduce expansion and accumulation	MCA26 colon cancer with liver metastases	mice	[89]
CK2 siRNA	caseine kinase 2 (CK2)-Notch signaling	improve differentiation and reduce expansion	EL4 lymphoma/CT26 colon carcinoma/Meth A sarcoma cell inoculation	mice	[92]
THC mediated DNA methylation	Arginase-1 and STAT3	promote differentiation and immunosuppressive function	none	mice	[44]
Histone deacetylase inhibition by TSA	Not mentioned	promote expansion	none	mice	[50]
HDAC11	Not mentioned	negative regulator of MDSC expansion and function	EL4 lymphoma cell inoculation	mice	[52]
HDAC2	Rb1	Phonotype switch	EL-4 thymoma, Lewis Lung Carcinoma (LLC) and 4T1 mammary carcinoma inoculation	mice	[54]

### Explore unknown mechanisms

Firstly, these studies are mainly implemented in murine models of diseases since murine MDSCs have been explicitly identified while human MDSCs have not. Whether these epigenetic modulatory approaches could actually work on patients requires further researches on accurate identification of human MDSCs and pre-clinical studies. Secondly, recent discussions on immunoregulatory mechanisms have focused on whether MSDC-mediated T cell suppression is antigen-specific or nonspecific. We favor that MDSC's suppressive effect on T-cell responses is in an antigen-specific manner because of the finding that T cells in the peripheral lymphoid organs of tumor-bearing mice as well as in the peripheral blood of cancer patients can still respond to stimuli other than tumor associated antigens [93-95]. Considering that G-MDSC derived ROS and peroxynitrite induce the nitration of TCR and result in its altered specific recognition of MHC/antigen epitopes, we hold the idea that it is G-MDSCs, though not as potent as M-MDSCs, that are more likely to perform specific inhibition of T cell response and are of greater meaning in the formation of MDSC-mediated tumor-specific tolerance in microenvironment. This implicates that the epigenetic approaches to re-orientate MDSC's differentiation toward M-type, though not inhibiting MDSCs directly, also have beneficial in prohibiting tumors. The last but not least, we wonder if other unreported types of epigenetic mechanisms also contribute to the altered characteristics of MDSCs, such as histone methylation and demethylation.

### Accelerate translating into clinical practice

For one thing, MDSC migration and function *in vivo*, as discussed above, could probably be modulated by more than one miRNA. Do these miRNAs action in the sole or overlapping signaling pathways? Is it possible that a panel of miRNAs modulate different checkpoints of certain cell process in a synergistic way? These questions remain to be answered for that two or more genes can be knocked down or silenced simultaneously by using a miRNA cocktail regimen. For another, the assessment on pharmacodynamics and pharmacokinetics is indispensable to determine whether or not these reagents could be used as drugs. The safety property of these reagents should also be taken into consideration for severe adverse effects are not permitted regardless of the therapeutic effect. With breakthroughs regarding human MDSCs, we believe the epigenetic modification on MDSCs could benefit specific and effective treatment for cancers in time.
